# Different Biological Action of Oleic Acid in ALDH^high^ and ALDH^low^ Subpopulations Separated from Ductal Carcinoma *In Situ* of Breast Cancer

**DOI:** 10.1371/journal.pone.0160835

**Published:** 2016-09-02

**Authors:** Hoe Suk Kim, Minji Jung, Sul Ki Choi, Woo Kyung Moon, Seung Ja Kim

**Affiliations:** 1 Department of Radiology, Seoul National University Hospital, 101 Daehak-ro, Jongno-gu, Seoul, Korea; 2 Department of Biomedical Science, College of Medicine, Seoul National University, 103 Daehak-ro, Jongno-gu, Seoul, Korea; 3 Department of Radiology, Sheikh Khalifa Specialty Hospital, Ras Al Khaimah, Abu Dhabi, United Arab Emirates; University of Alabama at Birmingham, UNITED STATES

## Abstract

The mechanisms underlying breast cancer progression of ductal carcinoma in situ **(**DCIS) associated with fatty acids are largely unknown. In the present study, we compared the action of oleic acid (OA) on two human DCIS cell lines, MCF10DCIS.COM (ER/PR/HER2-negative) and SUM225 (HER2 overexpressed). OA led to a significant increase in proliferation, migration, lipid accumulation and the expression of lipogenic proteins, such as SREBP-1, FAS and ACC-1, in MCF10DCIS.COM cells but not SUM225 cells. The ALDH^high^ subpopulation analyzed by the ALDEFLUOR assay was approximately 39.2±5.3% of MCF10DCIS.COM cells but was small (3.11±0.9%) in SUM225 cells. We further investigated the different biological action of OA in the distinct ALDH^low^ and ALDH^high^ subpopulations of MCF10DCIS.COM cells. OA led to an increase in the expression of ALDH1A1, ALDH1A2 and ALDH1A3 in MCF10DCIS.COM cells. SREBP-1 and ACC-1 were highly expressed in ALDH^high^ cells relative to ALDH^low^ cells, whereas FAS was higher in ALDH^low^ cells. In the presence of OA, ALDH^high^ cells were more likely to proliferate and migrate and displayed significantly high levels of SREBP-1 and FAS and strong phosphorylation of FAK and AKT relative to ALDH^low^ cells. This study suggests that OA could be a critical risk factor to promote the proliferation and migration of ALDH^high^ cells in DCIS, leading to breast cancer progression.

## Introduction

Ductal carcinoma in situ **(**DCIS) is defined by the presence of abnormal cells originating from the terminal duct unit in the breast and is considered a putative precursor for invasive breast cancer [1,2]. DCIS of the breast is a heterogeneous disease with biological, histological and clinical differences [3–6]. Breast cancer stem-like cells (BCSCs) exhibiting a CD44+/CD24-/lin- phenotype as well as the expression and activity of aldehyde dehydrogenase 1 (ALDH1) are detected in DCIS [7,8]. CD44+/ALDH^high^ cells display enhanced metastatic behavior and therapeutic resistance [9]. A DCIS subpopulation with ALDH1 expression and activity is more frequent in basal-like than luminal tumors and is considered to be involved in an early phase of cancer progression and to be different in its biological behavior and risk factors [7,10–13].

Because a link between obesity and diverse cancers has been suggested, resident adipocytes that secrete fatty acid are considered one of the risk factors to promote cancer progression [14]. A high level of free fatty acids in obesity is involved in the development of inflammatory changes and is associated with enhanced cancer risk [14,15]. Oleic acid (OA) and palmitic acid (PA), that are released from adipose tissue, are two of the most abundant fatty acids present in serum and function as both an energy source and a signal for activating gene expression, death, survival, growth, migration and invasion in various experimental systems [16]. The mechanisms underlying the cancer risk of fatty acids are largely unknown and their action appears to be differentially cancer type- and context-dependent. Fatty acids modulate gene expression including lipogenic genes through transcriptional networks [17]. The high expression of lipogenic genes, such as sterol regulatory element-binding proteins (SREBPs), fatty acid synthase (FAS) and acetyl-CoA carboxylase 1 (ACC-1), appears early in oncogenesis, and lipid accumulation confers cell survival in epithelial stem-like cells in DCIS and promotes the transition of DCIS to invasive cancer [12,18]. The complex mechanisms underlying DCIS progression to invasive breast cancer associated with fatty acids remains unresolved. To date the role of OA associated with breast cancer risk and progression is a controversial issue; the different functions and mechanisms of OA, which is the most abundant unsaturated fatty acid in plasma, on the anti-cancer effect or cancer risk have been revealed according to tumor types, especially molecular subtypes of breast cancer [16,19–26]. With regard to ALDH1 and OA, the underlying mechanism of OA-mediated proliferation and migration in distinct DCIS subpopulations with ALDH expression and activity remains poorly understood. In the present study, we compared the effect of OA on proliferation and migration in two human DCIS cell lines, MCF10DCIS.COM (estrogen receptor; ER, progesterone receptor; PR and HER2-negative) and SUM-225 (HER2 overexpressed) cells, and investigated the different action of OA on the cellular behavior of the distinct subpopulations (ALDH^high^ and ALDH^low^) isolated from MCF10DCIS.COM cells.

## Materials and Methods

### Cell lines and culture

MCF10DCIS.COM (ER, PR and HER2-negative DCIS cell line), SUM225 (ER/PR-negative and HER2-overexpressed DCIS cell line) and MCF10CA1h (human epithelial breast carcinoma cells) cells were purchased from Asterand (Detroit, MI, USA). MCF10A cells, which are normal human epithelial cells, were purchased from American Type Culture Collection (ATCC) (Manassas, VA, USA). MCF-7 cells (human ER and PR positive and HER negative- breast cancer cell lines), HCC1954 cells (human HER2 overexpressed breast cancer cell lines), HCC1937 and MDA-MB-231 cells (human triple negative breast cancer cell lines) were purchased from the Korean Cell Line Bank (KCLB) (Seoul, Korea) and the fingerprinting of cell lines by AmplFLSTR identifiler PCR Amplification kit was tested by the KCLB.

MCF10A cells were maintained in Dulbecco’s Modified Eagle Medium (DMEM)/F-12 (Invitrogen, Carlsbad, CA, USA) containing 5% horse serum (Invitrogen), 20 ng/ml EGF, 0.5 μg/ml hydrocortisone, 100 ng/ml cholera toxin, 10 μg/ml insulin (Sigma, St. Louis, MO, USA) and 1% penicillin/streptomycin. MCF10DCIS.COM, SUM225 and MCF10ACA1h cells were maintained in DMEM:F-12 (Gibco, Grand Island, NY, USA) containing 10% FBS, 2 mM L-glutamine (Sigma), and 1% penicillin**/**streptomycin. SUM225 cells were maintained in Ham’s F-12 (Gibco) containing 10% FBS, 5 μg/ml insulin (Sigma), 5 μg/ml hydrocortisone, 10mM HEPES, and 1% penicillin**/**streptomycin. MCF7, MDA-MB-231, HCC1937 and HCC1954 cells were maintained in RPMI-1640 (WelGENE, Seoul, Korea) containing 10% FBS, 2 mM L-glutamine, and 1% penicillin**/**streptomycin. All cell lines were grown at 37°C with 5% CO_2_ in tissue culture plates and passaged in our laboratory for fewer than 6 months after receipt or resuscitation.

### Cell viability

All cells were seeded in 96-well plates at 10^4^ cells per well. Cells were incubated with OA (0.05–0.2 mM) or PA (0.25–1 mM) for 72 h and treated with a PI3K/AKT inhibitor (10 μM LY294002) (Cell Signaling Technology, Danvers, MA, USA), MEK/ERK1/2 inhibitor (10 μM PD98059) (Cell Signaling Technology) or FAK inhibitor (10 μM PF573228) (Cell Signaling Technology). Cell viabilities were assessed by a standard 3-(4, 5-methylthiazol-2-yl)-2, 5-diphenyl-tetrazolium bromide (MTT) assay (Sigma). In brief, the medium was replaced with MTT-containing medium (0.5 mg/ml) and incubated at 37°C for 2 h, a solution of dimethyl sulfoxide was added, and the plate was incubated at 37°C overnight to solubilize formazon crystals. The plates were kept on a rocker shaker for 10–30 min at 26°C, and then absorbance was measured at 540 nm using a spectrophotometer (GE Healthcare, Piscataway, NJ, USA). The viabilities were expressed as the relative ratio to untreated cells, which represent the control.

### In vitro migration assays

To assess cell migration, 1 x 10^5^ cells were suspended in medium with 2% FBS and deposited into the upper chambers of a trans-well plate with 8.0 μm pore size (BD Biosciences, Franklin Lakes, NJ, USA). The lower chambers were filled with medium supplemented with 2% FBS and 0.1 mM OA or 0.25 mM PA in the presence or absence of 10 μM LY294002, 10 μM PD98059 or 10 μM FAK PF573228. Cells were incubated for 72 h at 37°C, and the migrated cells in the bottom chamber were stained with crystal violet solution (0.5% crystal violet in 20% methanol) for 10 min. Unbound crystal violet was removed by rinsing with distilled water, and crystal violet-stained cells were subsequently air-dried. Next, crystal violet was eluted from cells with 1% sodium dodecyl sulfate solution. The absorbance of crystal violet was measured at 550 nm using a spectrophotometer (GE Healthcare). The migration abilities were expressed as the relative ratio to untreated cells, which represent the control.

### Wound healing assay

Cells were seeded in six-well plates and cultured under permissive conditions until 90% confluence. After 24 h, the confluent cell monolayer was lightly and quickly scratched with a pipette tip to produce a straight line. The debris was removed, and the edge of the scratch was smoothed with phosphate buffered saline (PBS). The wound healing assays were performed in medium supplemented with 0.1 mM OA for 16 h. Images of cells were acquired with a microscope (Leica, Wetzlar, Germany) equipped with a CCD camera (Leica), and lateral migration activity was evaluated as the area of cells entering the rectangle.

### Flow cytometry analysis

Flow cytometry was performed using a FACScan instrument (BD Biosciences). MCF10DCIS.COM cells were dissociated and washed once in PBS containing 1–2% bovine serum albumin and 5 mM EDTA; the cells were subsequently stained with anti-CD24-APC (Invitrogen) and anti-CD24-PE (Invitrogen) at a concentration of 10 μl of antibody per 10^6^ cells before being incubated at 4°C for 15–30 min. Cells were washed once with PBS, and flow cytometry was performed to detect CD44+/CD24− cells using each fluorescence channel. Gates were determined by analyzing the unstained cells and single stains. Data for 10,000 cells were collected and analyzed using Cell Quest software version 3.3 (BD Biosciences).

### ALDEFLUOR assay and separation of ALDH^high^ and ALDH^low^ cells

The ALDEFLUOR reagent system (Stem Cell Technologies, Vancouver, Canada) was used in all experiments and served as an immunofluorescence method to detect the intracellular enzymatic activity of ALDH1. BODIPY-aminoacetaldehyde diethyl acetal (BAAA-DA) was treated with 2 M HCl and converted into BODIPY-aminoacetaldehyde (BAAA), which is a fluorescent substrate for ALDH1. Cells were treated as follows: 0.2–1.0 × 10^6^ cells were incubated in 1 ml of ALDEFLUOR assay buffer with a BAAA concentration of 1.5 μM for 45 min at 37°C. For each experiment, a sample of cells was stained under identical conditions with 50 μmol/l of the specific ALDH1 enzyme inhibitor diethylaminobenzaldehyde (DEAB; Sigma), which served as a negative control. ALDEFLUOR fluorescence was detected using the green fluorescence channel (530 ± 15 nm). Cells were kept on ice and separated into either ALDH^high^ or ALDH^low^ populations by a FACS Vantage SE cell sorter (BD Biosciences).

### Real-time RT-PCR analysis

Total RNA was extracted from cultured cells using TRIzol Reagent (Invitrogen). RNA quantity and quality were determined using a NanoDrop spectrophotometer (Thermo Fisher Scientific Inc., Waltham, MA USA). cDNA was produced using SuperScript II reverse transcriptase (Invitrogen). The following primers for ALDH1A1, ALDH1A2, ALDH1A3, and β-actin were used: ALDH1A1, 5′-TGTTAGCTGATGCCGACTTG-3′, and 5′- TTCTTAGCCCGCTCAACACT-3′; ALDH1A2, 5′-CTGGCAATAGTTCGGCTCTC-3′, and 5′-TGATCCTGCAAACACTGCTC-3′; ALDH1A3, 5′- TCTCGACAAAGCCCTGAAGT-3′, and 5′-TATTCGGCCAAAGCGTATTC-3′; and β-actin, 5′-TTCCTGGGCATGGAGTCCTG-3′ and 5′-CGCCTAGAAGCATTTGCGGT-3′. Real-time PCR reactions were run on an ABI PRISM^®^ 7900 using a SYBR Green PCR master mix (Applied Biosystems, Foster City, CA, USA). The results were analyzed by the ΔCt method, which reflects the difference in threshold for the target gene relative to that of β-actin in each sample.

### Western blot

The cells were lysed in RIPA buffer containing a protease inhibitor cocktail (Sigma), and the proteins were separated by SDS-PAGE and transferred to nitrocellulose membranes. The membranes were blocked with 5% skim milk in Tris-buffered saline and incubated with primary antibodies against phosphorylated-ERK1/2 (Cell Signaling Technology), phosphorylated-AKT (ser437) (Cell Signaling Technology), phosphorylated-FAK (Cell Signaling Technology), ERK (Cell Signaling Technology), AKT (Cell Signaling Technology), FAK (Cell Signaling Technology), SREBP-1 (Santa Cruz Biotechnology, Santa Cruz, CA, USA), FAS (Santa Cruz Biotechnology), ACC-1 (Santa Cruz Biotechnology) and β-actin (Sigma) overnight at 4°C, followed by incubation with horseradish peroxidase-conjugated secondary antibody (Santa Cruz Biotechnology) at room temperature for 30 min. The blots were developed using Enhanced Chemiluminescence Reagents (Amersham Biosciences, Piscataway, NJ, USA). The relative intensity of the bands observed by Western blotting was analyzed using the Image J program.

### Immunofluorescence staining

Cells were cultured on eight-well chamber slides, rinsed in PBS and fixed with 2% paraformaldehyde for 30 min at 4°C. The fixed cells were blocked with 2% bovine serum albumin in PBS; reacted with primary antibodies for ALDH1 (Sigma), CD44 (Sigma) and CD24 (Sigma); visualized using secondary antibodies conjugated to Alexa 594 and 488 (Invitrogen); and counterstained with 4',6-diamidino-2-phenylindole (DAPI). These fluorescently stained cells were then observed under a fluorescence microscope using LAS software (Leica) for image acquisition.

### Oil Red O staining

The cells were treated with 0.1 mM OA for 72 h. Cell were washed twice with PBS, fixed in 2% paraformaldehyde for 30 min at 4°C, stained for 1 h at room temperature with Oil Red O solution (Sigma) to detect intracellular lipid vacuoles, and then visualized using light microscopy. The dye content was extracted in isopropanol by shaking for 15 min at room temperature and quantified with spectrophotometry at 510 nm.

### Data analysis

All data were presented as the mean ± standard errors from at least three independent experiments and were analyzed using a t-test to test the difference in two groups. *P* values less than 0.05 were considered statistically significant.

## Results

### OA strongly promoted the proliferation and migration of MCF10DCIS.COM cells among diverse breast cancer cells

**Fig 1:**
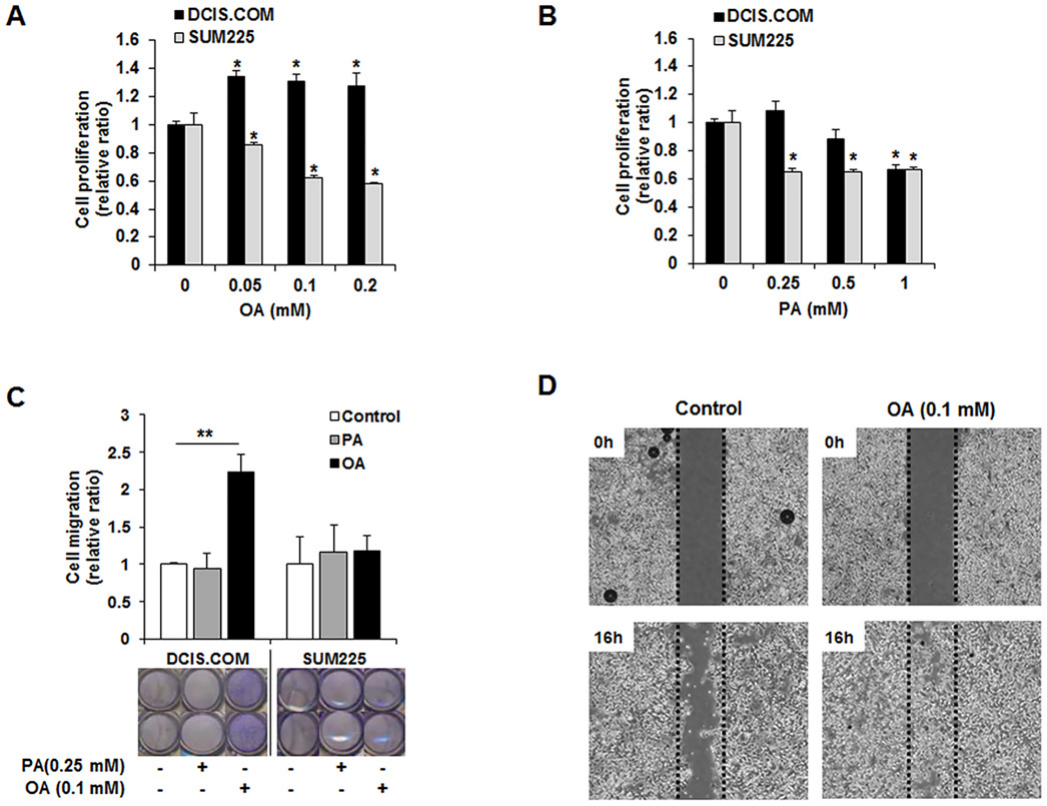
Oleic acid (OA) promotes the proliferation and migration ability of MCF10DCIS.COM cells but not SUM225 cells, whereas palmitic acid (PA) leads to cell death in both cells. (A and B) MTT assay of cell proliferation in MCF10DCIS.COM and SUM225 cells incubated with increasing OA or PA. OA induced significantly increased viability in MCF10DCIS.COM cells but led to cell death in SUM225 cells. PA induced the death of both MCF10DCIS.COM and SUM225 cells. (C) Trans-well assay of cell migration in MCF10DCIS.COM and SUM 225 cells. OA significantly promoted the migration of MCF10DCIS.COM cell but not SUM225 cells. (D) Wound healing assay of lateral migration of MCF10DCIS.COM cells incubated with OA. OA significantly enhanced the lateral migration of MCF10DCIS.COM cells. All the experiments were performed at least in triplicate and the values are reported as the means ± standard error. *p<0.05, **p<0.01.

As the first step to clarify the effects of OA and PA on cell proliferation and migration abilities, human breast epithelial cells (MCF10A) and diverse human breast cancer cell lines (MCF10CA1 h, MCF10DCIS.COM, SUM-225, MCF-7, HCC1954, HCC1937, MDA-MB-231) were evaluated by MTT and trans-well migration assays. Among diverse breast cancer cell lines, only MCF10DCIS.COM cells displayed increased proliferation and migration by treatment with 0.1 mM OA (S1 Fig). OA treatment significantly suppressed cell proliferation and migration in SUM225 and HCC1954 cells, which are HER2-overexpessed breast cancer cell lines (S1 Fig). When comparing the proliferation ability between MCF10DCIS.COM cells and SUM225 cells, OA (0.05 mM—0.2 mM) led to a significant increase in the proliferative capacity of only MCF10DCIS.COM cells, up approximately 1.4-fold relative to control cells after 72 h, but induced the cell death of SUM225 cells in a dose-dependent manner (*p<0.05, Fig 1A). PA induced the cell death of both cells dose-dependently (*p<0.05, Fig 1B). The migratory abilities of MCF10DCIS.COM and SUM225 cells incubated with and without OA or PA for 16 h were assessed by 8 μm pore trans-well chambers. The migrating MCF10DCIS.COM cells increased more than 2-fold by treatment with 0.1 mM OA but not 0.25 mM PA (**p<0.01, Fig 1C). However, the migratory ability of SUM225 cells was not affected by OA or PA at similar concentrations. Wound healing to monitor lateral migration was assayed in MCF10DCIS.COM cells; 0.1 mM OA significantly enhanced the lateral migration ability of MCF10DCIS.COM cells (Fig 1D).

### OA differentially regulated lipogenic genes in MCF10DCIS.COM and SUM225 cells

**Fig 2:**
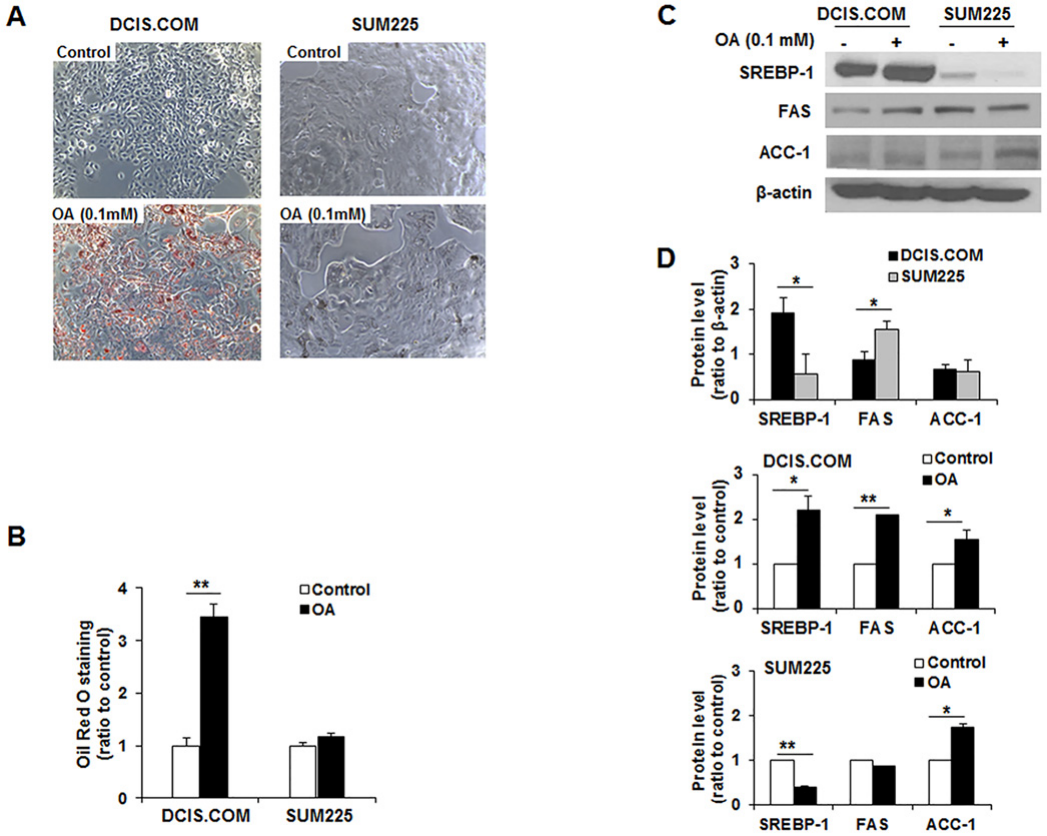
Oleic acid (OA) induces lipid accumulation and the upregulation of lipogenic proteins in MCF10DCIS.COM cells but not SUM225 cells. (A) Representative Oil Red O staining in MCF10DCIS.COM and SUM 225 cells incubated with OA. A large number of lipid droplets were observed in MCF10DCIS.COM cells but not SUM225 cells. (B) Quantitative analysis of intracellular lipid contents from Oil Red O staining. OA led to lipid accumulation in MCF10DCIS.COM cells. (C) Representative Western blot of SREBP-1, FAS and ACC-1 in MCF10DCIS.COM and SUM 225 cells incubated with OA. (D) Quantitative analysis of lipogenic protein levels in MCF10DCIS.COM and SUM225 cells. A significantly higher level of SERBP-1 was observed in MCF10DCIS.COM cells relative to SUM225 cells. The FAS level was significantly higher in SUM225 cells than MCF10DCIS.COM cells. The level of ACC-1 was similar between MCF10DCIS.COM cells and SUM225 cells (upper). OA resulted in the significant upregulation of SREBP-1, FAS and ACC-1 in MCF10DCIS.COM cells (middle) but led to the downregulation of SREBP-1 and the upregulation of ACC-1 significantly in SUM225 cells (lower). All experiments were performed at least in triplicate, and the values are reported as the means ± standard error. *p<0.05, **p<0.01.

Next, we investigated lipid droplet accumulation through supplementation with OA for 72 h in MCF10DCIS.COM and SUM225 cells. Oil Red O staining showed a large amount of intracellular lipid droplets in OA-treated MCF10DCIS.COM cells but not SUM225 cells (Fig 2A). The treatment of 0.1 mM OA in MCF10DCIS.COM cells resulted in a 3-fold increase by quantitative analysis of Oil Red O staining (**p<0.01, Fig 2B). To determine the expression levels of SREBP-1, FAS and ACC-1, which are involved in lipogenesis, proliferation and survival of cancer cells, the levels of these proteins was assessed by Western blot (Fig 2C). When compared to SREBP-1, FAS and ACC-1 levels between MCF10DCIS.COM and SUM225 cells, the SREBP-1 level was higher in MCF10DCIS.COM cells than SUM225 cells (1.91 ± 0.17 vs 0.57 ± 0.17, *p<0.05, Fig 2D top). The FAS level was higher in SUM225 cells than MCF10DCIS.COM cells (1.56 ± 0.17 vs 0.88 ± 0.17, *p<0.05, Fig 2D top). The expression level of ACC-1 was similar between MCF10DCIS.COM cells and SUM225 cells (Fig 2D top). SREBP-1, FAS and ACC-1 were significantly increased to 2.22 ± 0.29, 2.09 ± 0.002 and 1.53± 0.15, respectively, in OA-treated MCF10DCIS.COM cells relative to the control (*p<0.05, **p<0.01, Fig 2D middle). OA-treated SUM225 cells exhibited a significant increase in ACC-1 (1.73 ± 0.09) and a significant decrease in SREBP-1 (0.42 ± 0.005) compared to the control (*p<0.05, **p<0.01, Fig 2D bottom).

### OA stimulated cell proliferation and migration through the FAK, PI3K/AKT and MAPK-mediated signaling pathway in MCF10DCIS.COM cells

**Fig 3:**
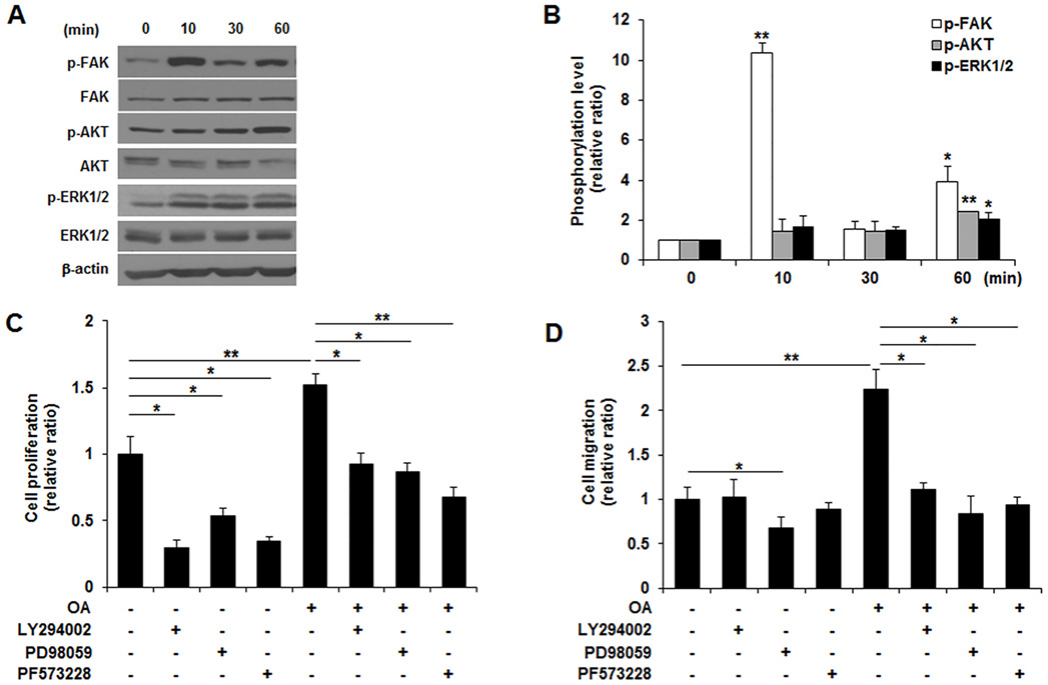
Oleic acid (OA) promotes the viability and migration through the FAK, PI3K/AKT, and MEK/ERK signaling pathway in MCF10DCIS.COM cells. (A and B) Representative Western blot and quantitative analysis of phosphorylated FAK, AKT and ERK1/2 in MCF10DCIS.COM cells incubated with OA. OA induced a significant increase in the phosphorylation of FAK, AKT and ERK1/2. (C) MTT assay of cell proliferation in MCF10DCIS.COM cells incubated with OA in the presence of FAK (PF573228), PI3K/AKT (LY294002) and MEK/ERK (PD98059) inhibitors. All kinase inhibitors induced cell death, and OA-promoted proliferation was reduced in the presence of all kinase inhibitors. (D) Trans-well assay of cell migration in MCF10DCIS.COM cells incubated with OA in the presence of FAK, PI3K/AKT and MEK/ERK inhibitors. OA-induced migration was suppressed by the presence of all kinase inhibitors. All the experiments were performed at least in triplicate, and the values are reported as the means ± standard error. *p<0.05, **p<0.01.

The involvement of the FAK, PI3K/AKT and MAPK-mediated signaling pathway in OA-induced proliferation and migration in MCF10DCIS.COM cells was examined. OA promoted the phosphorylation of FAK, AKT and ERK in a time-dependent manner (*p<0.05, **p<0.01, Fig 3A and 3B). Pretreatment with potent chemical inhibitors of PI3K (LY294002, 20μM), MEK (PD98059, 20μM), or FAK (PF573228, 5μM) caused cell death and significantly suppressed OA-induced cell proliferation ability (*p<0.05, **p<0.01, Fig 3C). OA-enhanced migration ability was also inhibited by pretreatment with PI3K, MEK and FAK inhibitors, and the result was statistically significant (*p<0.05, Fig 3D).

### MCF10DCIS.COM cells contained distinct subpopulations of ALDH1^high^ and ALDH1^low^ cells

**Fig 4:**
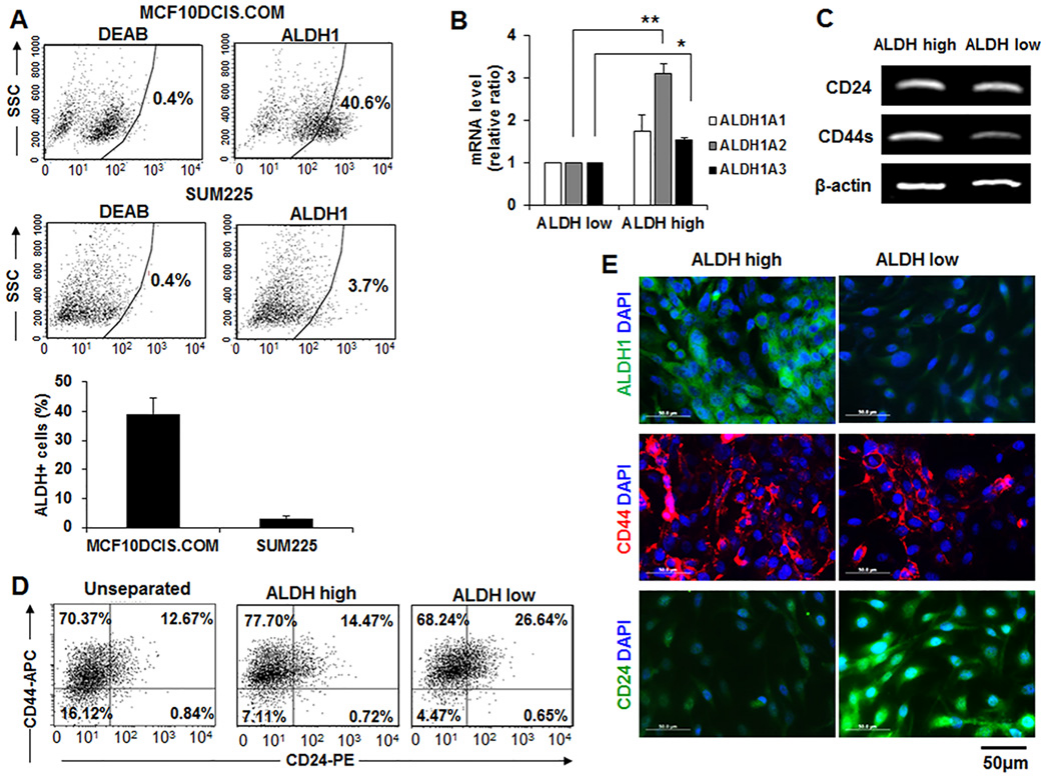
Distinct subpopulations of ALDH1^high^ and ALDH1^low^ cells were separated from MCF10DCIS.COM cells. (A) Representative flow cytometry for ALDEFLUOR assay showing the percentage of ALDH^high^ cells in MCD10DCIS.COM and SUM225 cells. Graph showed that the ALDH^high^ cell population obtained from experiments performed at least in triplicate. Cells exhibiting high ALDH activity were higher in MCF10DCIS.COM cells relative to SUM225 cells. (B) Quantitative real-time RT-PCR of ALDH1A1, ALDH1A2 and ALDH1A3 in ALDH1^high^ and ALDH1^low^ subpopulation cells separated from MCF10DCIS.COM cells. Significantly higher expression levels of ALDH1A2 and ALDH1A3 mRNAs were detected in ALDH^high^ cells relative to ALDH^low^ cells. The experiments were performed at least in triplicate, and the values are reported as the means ± standard error. *p<0.05, **p<0.01. (C) RT-PCR analysis of CD24 and CD44 mRNAs in ALDH^high^ and ALDH^low^ cells. CD44 mRNA was higher in ALDH1^high^ cells than ALDH1^low^ cells. (D) Flow cytometric analysis of CD44 and CD24. Of MCF10DCIS.COM cells, 70% exhibited the CD44+/CD24- phenotype. CD44+/CD24- cell populations were higher in separated ALDH1^high^ cells than ALDH1^low^ cells. (E) Immunofluorescence staining of CD44, CD24 and ALDH1. ALDH1^high^ cells expressed a high level of ALDH1 and CD44, whereas ALDH1^low^ cells displayed a high level of CD24 and a low level of ALDH1 and CD44.

Flow cytometry based on the ALDEFLUOR assay for the measurement of ALDH1 activity showed that ALDH1-positive cells were approximately 39.2±5.3% of the MCF10DCIS.COM cells and (3.11±0.9% of SUM225 cells (Fig 4A). Our data indicate the presence of a large, clearly distinguishable subpopulation of ALDH-positive cells in MCF10DCIS.COM cells compared to SUM225 cells. ALDH^high^ and ALDH^low^ cells were separated from MCF10DCIS.COM cells, and the splice variants of ALDH1 subtypes, ALDH1A1, ALDH1A2 and ALDH1A3, were compared in both subpopulations. ALDH1A1, ALDH1A2 and ALDH1A3 increased to 1.75±0.38, 3.11±0.23 (**p<0.01) and 1.52±0.07 (*p<0.05), respectively in ALDH^high^ cells relative to ALDH^low^ cells (Fig 4B). The CD44 transcripts were higher in ALDH^high^ cells than ALDH^low^ cells (Fig 4C). The phenotype of CD44 and CD24 in ALDH^high^ and ALDH^low^ cells was further analyzed by flow cytometry. A large subpopulation was CD44+/CD24- (70.37%) in unsorted MCF10DCIS.COM cells (Fig 4D left). Compared to the phenotypes between ALDH^high^ cells and ALDH^low^ cells separated from MCF10DCIS.COM cells, CD44+/CD24- cells were higher in ALDH^high^ cells (77.7%) than ALDH^low^ cells (68.24%) (Fig 4D). CD44, CD24 and ALDH1 expression were further investigated by immunostaining. ALDH^high^ cells expressed a high level of ALDH and CD44, whereas ALDH^low^ cells expressed a high level of CD24 (Fig 4E).

### OA led to a significant increase in the expression of ALDH1 transcripts in MCF10DCIS.COM cells and ALDH^high^ cells responding to OA exhibited higher proliferation and migration rates than ALDH^low^ cells

**Fig 5:**
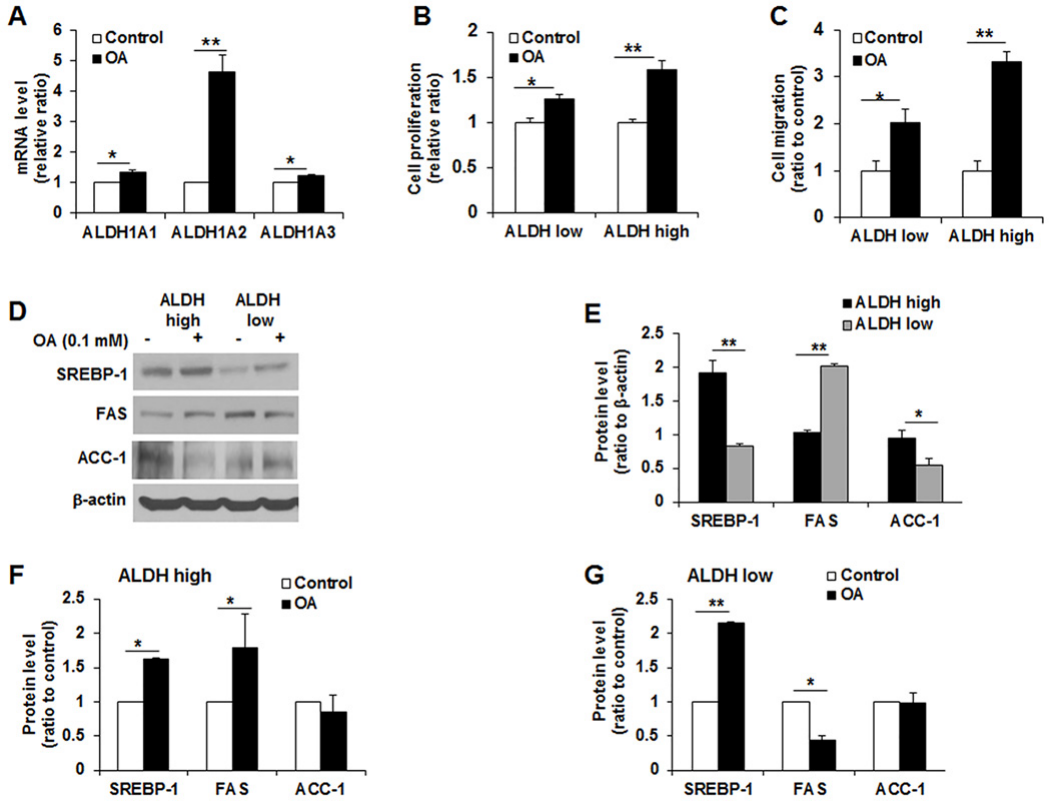
Oleic acid (OA) further promotes the proliferation and migration abilities and upregulates lipogenic proteins in ALDH^high^ cells. (A) Quantitative real-time RT-PCR of ALDH1A1, ALDH1A2 and ALDH1A3 in MCF10DCIS.COM cells. All subtypes of ALDH1 were significantly increased by OA. Notably, OA led to a remarkable increase in ALDH1A2 in MCF10DCIS.COM cells. (B) MTT assay of cell proliferation in ALDH^high^ and ALDH^low^ cells incubated with OA. OA-induced proliferation of ALDH^high^ cells was greater than that of ALDH^low^ cells. (C) Trans-well assay of cell migration in ALDH^high^ and ALDH^low^ cells. The OA-induced migration ability was higher in ALDH^high^ cells than ALDH^low^ cells. (D) Representative Western blot of SREBP-1, FAS and ACC-1 in ALDH^high^ cells and ALDH^low^ cells. (E, F and G) Analysis of expression levels of SREBP-1, FAS and ACC-1. Significantly higher expression of SERBP-1 and ACC-1 was observed in ALDH^high^ cells, whereas FAS was significantly higher in ALDH^low^ cells. OA led to the significant upregulation of SREBP-1 and FAS in ALDH^high^ cells and the significant upregulation of SREBP-1 and downregulation of FAS in ALDH^low^ cells. All experiments were performed at least in triplicate, and the values are reported as the means ± standard error. *p<0.05, **p<0.01.

OA significantly increased ALDH1A1, ALDH1A2 and ALDH1A3 transcripts in unseparated MCF10DCIS.COM cells, and the level of the ALDH1A2 transcript dramatically increased approximately 4-folds (3.9 ± 0.87) in OA-treated cells relative to control cells (*p<0.05, **p<0.01, Fig 5A). OA-modulated cell proliferation and migration abilities were compared between ALDH^high^ and ALDH^low^ cells. Cell proliferation was more strongly promoted by OA in ALDH^high^ cells (1.58 ± 0.09) than ALDH^low^ cells (1.27 ± 0.06) (*p<0.05, **p<0.01, Fig 5B). We tested the effect of OA on cell migration in ALDH^high^ and ALDH^low^ cells. As shown in Fig 5C,OA strongly increased the cell migration of ALDH^high^ cells (3.33 ± 0.19) relative to ALDH^low^ cells (2.02 ± 0.29) (*p<0.05, **p<0.01). A representative Western blot showed the different expression level of SREBP-1, FAS and ACC-1 in ALDH^high^ and ALDH^low^ cells (Fig 5D). The protein levels of SREBP-1 and ACC-1 in ALDH^high^ cells were approximately 2-fold higher than those in ALDH^low^ cells, and the FAS level was approximately 2-fold lower in ALDH^high^ cells compared to ALDH^low^ cells (*p<0.05, **p<0.01 Fig 5E). OA caused ALDH^high^ cells to significantly increase SREBP-1 (1.62 ± 0.01) and FAS (1.79 ± 0.48) but slightly decreased ACC-1 (0.85 ± 0.24) (*p<0.05, Fig 5F). After treatment with OA, ALDH^low^ cells exhibited a significant upregulation of SERBP-1 (2.15 ± 0.02) and downregulation of FAS (0.43 ± 0.07) (*p<0.05, **p<0.01, Fig 5G).

### OA-augmented proliferation and migration of ALDH^high^ cells were associated with the strong activation of FAK and AKT compared to ALDH^low^ cells

**Fig 6:**
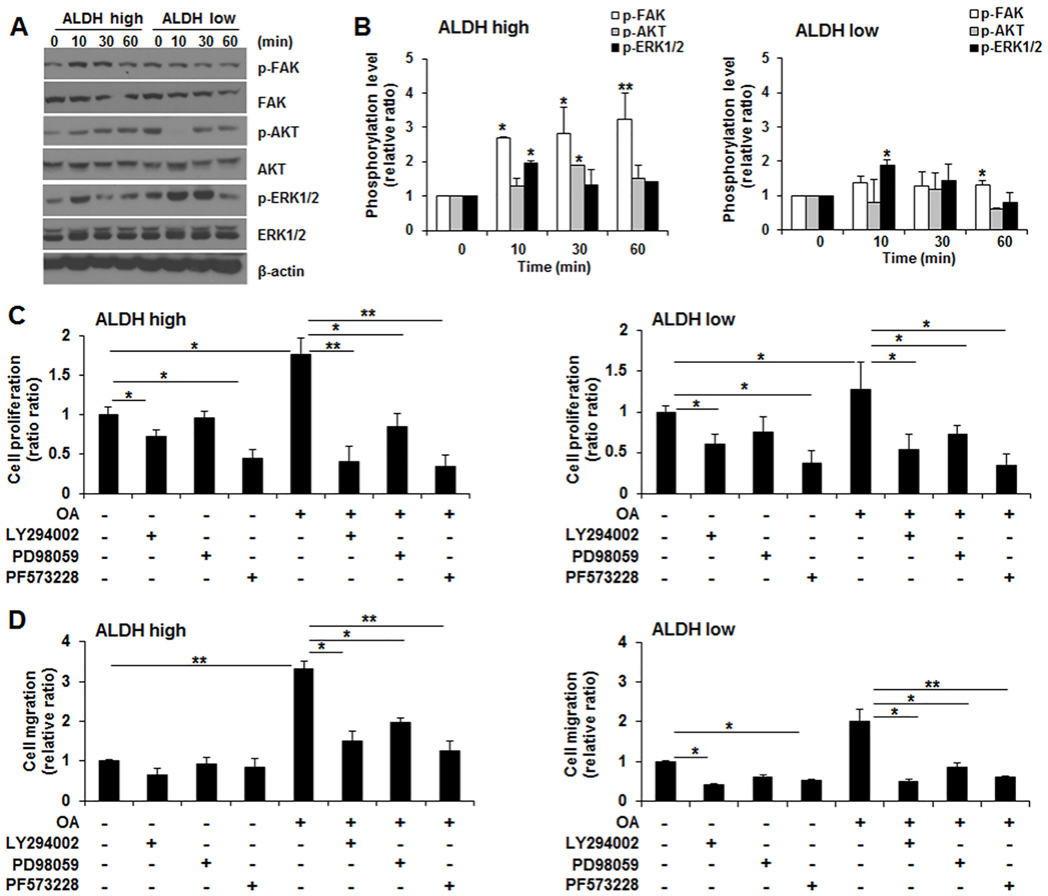
Oleic acid (OA) leads to the strong phosphorylation of FAK and AKT in ALDH^high^ cells, resulting in high proliferation and migration. (A) Representative Western blot of the phosphorylation of FAK, AKT and ERK1/2 in ALDH^high^ cells and ALDH^low^ cells treated with OA. (B) Analysis of the phosphorylated levels of FAK, AKT and ERK1/2 in ALDH^high^ and ALDH^low^ cells. The phosphorylation level of FAK and AKT was higher in ALDH^high^ cells compared to ALDH^low^ cells. (C) MTT assay of cell viability in the presence of FAK (PF573228), PI3K/AKT (LY294002) and MEK/ERK (PD98059) inhibitors. All inhibitors decreased the cell viability and suppressed the proliferative ability promoted by OA in ALDH^high^ and ALDH^low^ cells. (D) Trans-well assay of cell migration in ALDH^high^ cells and ALDH^low^ cells pretreated with FAK (PF573228), PI3K/AKT (LY294002) and MEK/ERK (PD98059) inhibitors. The OA-promoted migration was suppressed by the presence of all kinase inhibitors. All the experiments were performed at least in triplicate, and the values represent the means ± standard error. *p<0.05, **p<0.01.

To investigate the underlying mechanism of OA on the highly augmented proliferation and migration of ALDH^high^ cells compared to those of ALDH^low^ cells, the signaling pathways that are involved in cancer cell proliferation and migration were investigated. Fig 6A showed the OA-induced phosphorylation of FAK, AKT and ERK in ALDH^high^ and ALDH^low^ cells. The phosphorylation levels of FAK and AKT stimulated by OA time-dependently increased 2-4-fold in ALDH^high^ cells (*p<0.05, **p<0.01, Fig 6B left). ALDH^low^ cells exhibited an approximately 2-fold increase in the phosphorylation level of ERK in the presence of OA (*p<0.05, Fig 6B right). The cell viabilities in untreated ALDH^high^ and ALDH^low^ cells were significantly decreased by treatment with PI3K and FAK inhibitors, and the OA-induced proliferative activity in ALDH^high^ and ALDH^low^ cells was significantly suppressed by PI3K, MEK and FAK inhibitors (*p<0.05, **p<0.01, Fig 6C). The migration of ALDH^high^ cells incubated without OA was slightly decreased by all inhibitors, and the treatment of ALDH^high^ cells with PI3K and FAK inhibitors significantly suppressed the OA-augmented migration to a similar level of untreated ALDH^high^ cells (*p<0.05, **p<0.01, Fig 6D left). Adding the PI3K, MEK and FAK inhibitors significantly decreased migration in untreated ALDH^low^ cells and strongly reduced OA-augmented migration of ALDH^low^ cells under the basal levels obtained in the absence of OA (*p<0.05, **p<0.01, Fig 6F right).

## Discussion

Different types of free fatty acids act in numerous biological functions, such as cancer cell growth, death and migration, as a source of energy and many signaling molecules [16,27]. DCIS is believed to be a precursor of invasive breast cancer. Accordingly, the prevention of DCIS is considered the most effective way to reduce breast cancer progression and death. MCF10DCIS.COM and SUM225 cells are representative two human DCIS cell lines. The MCF10DCIS.COM cell line, which was shown to be reproducible from DCIS-like comedo lesions, is a clonal breast cancer cell line derived from a xenograft originating from premalignant MCF10AT cells, and SUM225 cells have amplified HER2 [28,29]. In present study, we demonstrated the different effect of OA and PA on the migration and viability of MCF10DCIS.COM cell and SUM225 cells. OA significantly increased the proliferative and migratory abilities in MCF10DCIS.COM cells not SUM225 cells, whereas PA induced the death of both cell types. In our study, OA did not promote the proliferation and migration of other breast cancer cells, MCF10CA1h, SUM225, MCF-7, HCC1354, HCC-1937 and MDA-MB-231 except for MCF10DCIS.COM cells. Interestingly, OA drastically induced cell death in SUM225 cells and suppressed the migration ability in HCC1954 cells, which are a representative HER2-overexpressed breast cancer cell line. Our results support that OA co-treatment with trastuzumab synergistically enhanced apoptotic cell death by promoting DNA fragmentation through caspase-3-dependent PARP cleavage; both the expression and nuclear accumulation of p27^Kip1^ and the inhibition of the AKT and MAPK signaling pathway play a key role in the onset and progression of HER2-related breast cancer in BT-474 and SKBR3 cells [30]. The inhibitory effect of PA on the proliferation of MCF10DCIS.COM cells was consistent with a report by Hardy *et al*. that PA inhibited cell proliferation and caused cell death of human triple negative MDA-MB-231 breast cancer cells [21,31]. Our result is inconsistent with other groups’ reports that OA promotes the proliferation and migration of MDA-MB-231 cells [23,24]. Inconsistent past results might be explained by differences in the environment of the experiments, which may cue a cell to respond positively to OA stimuli.

Increased lipogenesis and lipid accumulation appears early in oncogenesis and promotes the transition from pre- and high-risk lesions to invasive cancer in many types of tumors. Thus, the upregulation of lipogenic genes, such as SREBP1, FAS and ACC1, is a hallmark of cancer, and the overexpression of these genes is observed at an early stage in breast tumors [18,32]. Recently, elevated lipogenesis increased the cell survival of BCSC in DCIS [12]. In our study, OA treatment resulted in lipid accumulation in MCF10DCIS.COM cells but not SUM225 cells. This result suggests that lipid loading in MCF10DCIS.COM cells is one mechanism promoting cancer cell survival or invasive cancer progression. The overexpression of lipogenic genes is observed at an early stage of breast tumors [12,18,32]. SREBP-1 and one of its target genes, FAS, which is reported to be a metabolic oncogene, have been shown to be involved in malignant cancer progression, and SREBP-1 contributed to accumulating lipid droplets [18,33]. We found that MCF10DCIS.COM cells highly expressed SREBP-1, whereas SUM225 cells highly expressed FAS. Interestingly, OA induced the significant upregulation of SREBP-1 and FAS in MCF10DCIS.COM cells but led to the downregulation of both proteins in SUM225 cells. Taken together with previous reports, our results reflect that SREBP-1 elevated by OA contributes to lipid droplet accumulation and FAS upregulation in MCF10DCIS.COM cells.

DCIS of the breast is a heterogeneous disease with variable malignant potential, which enhances the complexity when attempting to define the mechanisms that promote progression to invasive cancer in an *in vivo* model [1,2,6]. The heterogeneity among DCIS within the same patient has not been well evaluated, leaving research implications of intra-individual DCIS heterogeneity yet to be explored [6,10]. MCF10DCIS.COM cells are suggested to be a unique model to study DCIS as well as the role of factors to regulate the progression to invasive breast cancer because they expressed a high level of basal markers (CD44 and CD49f), a low level of luminal markers (CD24 and MUC-1) and putative BCSC markers (ALDH1) and result in basal-like DCIS with high similarities to human DCIS samples [28,34,35]. ALDH1-positive cancer cells have been considered to mark breast epithelium at risk for breast cancer development and play a critical role in mediating the clinically aggressive behavior of breast cancer [10,11,13,36]. ALDH1 is suggested to be a predictor of early tumor relapse characteristics of invasive ductal carcinoma to contribute to aggressive behavior of breast cancer, self-renewal and proliferation [7,37]. In the present study, the distinct subpopulations of ALDH^low^ and ALDH^high^ cells, depending on ALDH1 activity and expression, were separated from MCF10DCIS.COM cells. ALDH^high^ cells exhibited a high level of ALDH1A1 and ALDH1A3 and represented the CD44+/CD24—phenotype compared to ALDH^low^ cells. Notably, OA induced a dramatic increase in ALDH1A2 expression in ALDH1^low^ cells but reduced expression in ALDH^high^ cells. More importantly, the proliferation and migration analyses identified that ALDH1^high^ cells were more likely to be proliferative and migratory in response to OA. Among lipogenic genes, the master regulator of lipogenesis, SREBP1, was abundantly expressed in epithelial stem-like cells in DCIS [12]. Supporting the previous report, OA led to a significant increase in the expression of genes including SREBP-1, CD44 and ALDH1, resulting in an increased BCSC subpopulation displaying CD44+/CD24-/ALDH1^high^. These results imply that OA may confer a survival advantage and cancer stem cell-like traits to MCF10DCIS.COM cells by upregulating lipogenic genes and BCSC markers.

The action of OA on breast cancer remains controversial. Some studies have suggested that OA exerts anti-tumorigenic effects by suppressing the expression of HER2 and boosts the effectiveness of trastuzumab [30]. On the other hand, the evidence that OA may influence breast cancer risk comes from other studies [19,23–25]. OA stimulated survival and proliferation through the activation of a free fatty acid receptor 1 (FFAR1)-dependent MAPK pathway and PI3K pathway, induced invasion and migration through MMPs and FAK-Src activation and reduced apoptosis in an aggressive breast cancer cell line, MDA-MB-231 cells [21,38]. The effect and underlying mechanisms of OA on the proliferation and migration activities of DCIS cells are not completely understood. We found that OA promoted AKT, ERK and FAK activation in MCF10DCIS.COM cells. Notably, ALDH^high^ cells separated from MCF10DCIS.COM cells displayed strong phosphorylation of FAK and AKT in the presence of OA relative to ALDH^low^ cells. The pretreatment of ALDH^high^ and ALDH1^low^ cells with chemical inhibitors resulted in a significant reduction in OA-augmented viability and migration. Our findings indicate that OA may contribute to DCIS progression to invasive breast cancer through the AKT-, ERK- and FAK-mediated signal pathways, which are considered to play a pivotal role in proliferation, migration and invasion in breast cancer [24,25]. Importantly, by evaluating the expression of the promising risk biomarker ALDH1 among the subpopulations of MCF10DCIS.COM cells, our findings suggest that OA is a high risk factor for the progression of ALDH^high^ DCIS into invasive breast cancer. Overall, we observed the strong action of OA in the proliferation and migration of ALDH1-positive DCIS cells among different subtypes of breast cancer cells. Our findings suggest that OA might be a critical risk factor to promote the progression of DCIS to invasive breast cancer, and the ALDH^high^ subpopulation with BCSC characteristics expressing a high level of ALDH1 and CD44 in DCIS of breast cancer may contribute to early invasion and migration in response to OA, possibly in association with the FAK and PI3K/AKT signaling pathway. OA activity and ALDH1 status should be further investigated in detail as a potential prognostic and therapeutic target to prevent the transition from DCIS to invasive breast cancer in translating DCIS research into clinical practice. Therefore, investigating the action of OA in the distinct ALDH^high^ and ALDH^low^ subpopulations of DCIS may help to provide insight into the biologic heterogeneity of breast cancer progression. Our study suggests that there is a need for a more tailored approach to basic research as well clinical treatment because DCIS is a very heterogeneous disease.

## Supporting Information

S1 Fig Oleic acid (OA) leads to a significant increase in viability and migration in MCF10DCIS.COM cells compared with other breast cancer cell lines. (A) Cell viability in diverse breast cancer cells incubated with 0.1 mM OA. The viability was significantly increased in MCF10DCIS.COM cells but rather decreased in SUM225 and MDA-MB-231 cells after treatment with OA. The change in cell viability was not observed in other cell lines incubated with OA. (B) Cell migration ability in diverse breast cancer cells incubated with 0.1 mM OA was subjected to trans-well assay. OA increased migration in MCF10DCIS.COM cells but decreased migration in HCC1954 cells. All the experiments were performed at least in triplicate, and the values represent as the means ± standard error. *p<0.05, **p<0.01.
(TIF)
