# Supplementary figures and images for: Different Biological Action of Oleic Acid in ALDHhigh and ALDHlow Subpopulations Separated from Ductal Carcinoma In Situ of Breast Cancer
Source: PLoS One. 2016 Sep 2;11(9):e0160835. doi: 10.1371/journal.pone.0160835 (PMC5010246; doi:10.1371/journal.pone.0160835)

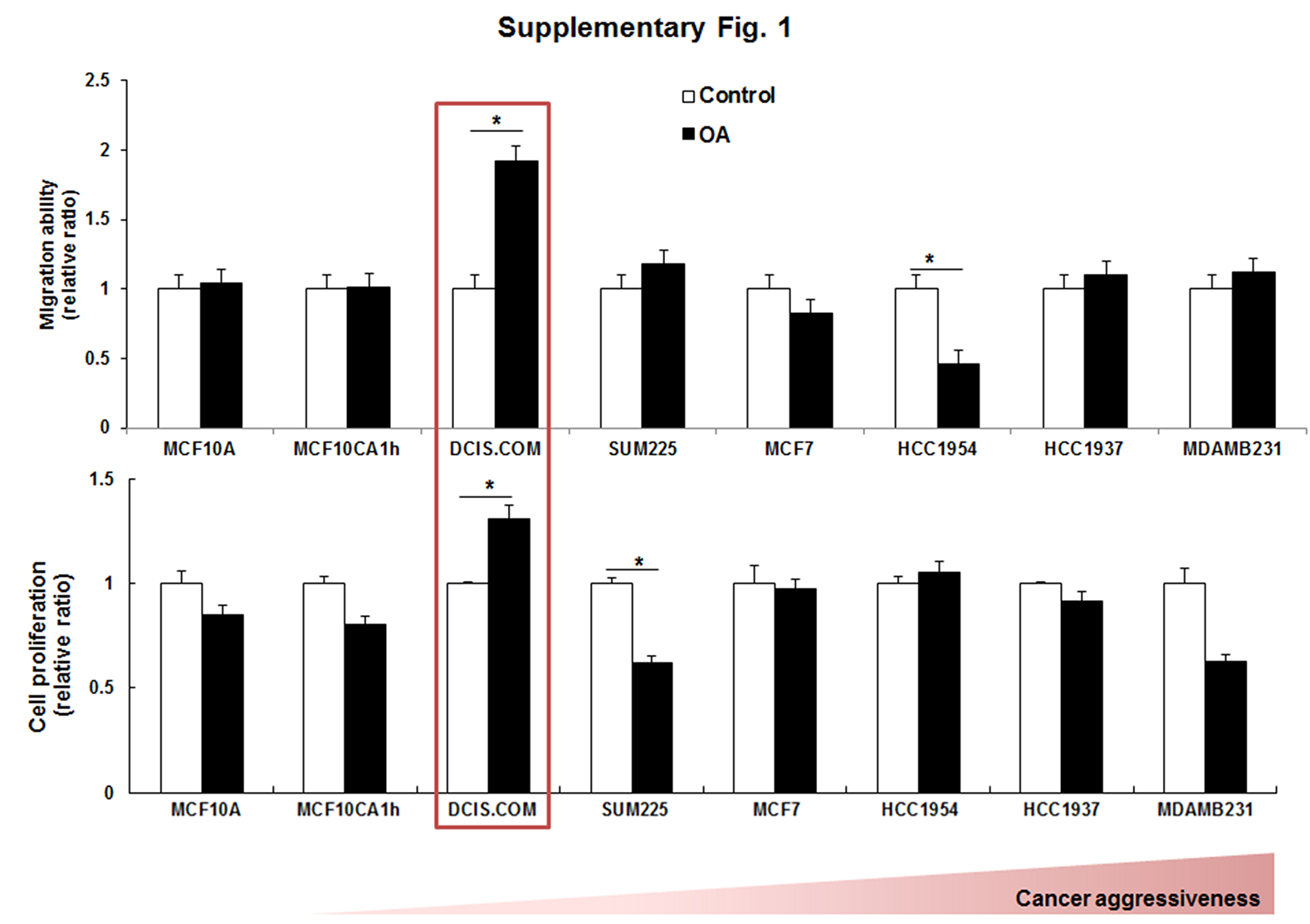

Supplement: S1 Fig — (A) Cell viability in diverse breast cancer cells incubated with 0.1 mM OA. The viability was significantly increased in MCF10DCIS.COM cells but rather decreased in SUM225 and MDA-MB-231 cells after treatment with OA. The change in cell viability was not observed in other cell lines incubated with OA. (B) Cell migration ability in diverse breast cancer cells incubated with 0.1 mM OA was subjected to trans-well assay. OA increased migration in MCF10DCIS.COM cells but decreased migration in HCC1954 cells. All the experiments were performed at least in triplicate, and the values represent as the means ± standard error. *p<0.05, **p<0.01. (TIF) [file pone.0160835.s001.tif]
